# *Curcuma longa* Extract Associated with White Pepper Lessens High Fat Diet-Induced Inflammation in Subcutaneous Adipose Tissue

**DOI:** 10.1371/journal.pone.0081252

**Published:** 2013-11-19

**Authors:** Audrey M. Neyrinck, Maud Alligier, Patrick B. Memvanga, Elodie Névraumont, Yvan Larondelle, Véronique Préat, Patrice D. Cani, Nathalie M. Delzenne

**Affiliations:** 1 Université catholique de Louvain, Louvain Drug Research Institute, Metabolism and Nutrition Research Group, Brussels, Belgium; 2 Université catholique de Louvain, Louvain Drug Research Institute, Pharmaceutics and Drug delivery Group, Brussels, Belgium; 3 Université catholique de Louvain, Institut des Sciences de la Vie, Louvain-la-Neuve, Belgium; Instutite of Agrochemistry and Food Technology, Spain

## Abstract

**Background:**

Supra-nutritional doses of curcumin, derived from the spice *Curcuma longa*, have been proposed as a potential treatment of inflammation and metabolic disorders related to obesity. The aim of the present study was to test whether *Curcuma longa* extract rich in curcumin and associated with white pepper (Curcuma-P®), at doses compatible with human use, could modulate systemic inflammation in diet-induced obese mice. We questioned the potential relevance of changes in adiposity and gut microbiota in the effect of Curcuma-P® in obesity.

**Methodology/Principal Findings:**

Mice were fed either a control diet (CT), a high fat (HF) diet or a HF diet containing *Curcuma longa* extract (0.1 % of curcumin in the HF diet) associated with white pepper (0.01 %) for four weeks. Curcumin has been usually combined with white pepper, which contain piperine, in order to improve its bioavailability. This combination did not significantly modify body weight gain, glycemia, insulinemia, serum lipids and intestinal inflammatory markers. Tetrahydrocurcumin, but not curcumin accumulated in the subcutaneous adipose tissue. Importantly, the co-supplementation in curcuma extract and white pepper decreased HF-induced pro-inflammatory cytokines expression in the subcutaneous adipose tissue, an effect independent of adiposity, immune cells recruitment, angiogenesis, or modulation of gut bacteria controlling inflammation.

**Conclusions/Significance:**

These findings support that nutritional doses of *Curcuma longa*, associated with white pepper, is able to decrease inflammatory cytokines expression in the adipose tissue and this effect could be rather linked to a direct effect of bioactive metabolites reaching the adipose tissue, than from changes in the gut microbiota composition.

## Introduction

The high prevalence of obesity is a major threat to the public’s health. Obesity is associated with substantially decreased health-related quality of life, and increased medical expenses [1]. Given the large burden imposed by obesity on the welfare of society, it is imperative to develop new methods that can decrease its prevalence as well as reverse its detrimental physiological alterations. Growing evidence suggests that obesity and related metabolic disorders such as insulin resistance, type 2 diabetes, and cardiovascular diseases are associated with a low-grade inflammatory state [2]. Several data suggest that the activity of the gut microbiota is an important factor to take into account when assessing the risk factors related to obesity, and associated disorders, such as dyslipidemia, inflammation, insulin resistance and diabetes [3-6]. Indeed, alterations in the composition of gut microbiota -known as dysbiosis- has been proposed to contribute to the development of obesity, thereby supporting the potential interest of nutrients acting on the gut microbes to produce beneficial effect on host energy metabolism. We and others have demonstrated the interest to modulate the gut microbiota composition by dietary carbohydrates called prebiotics, which allow decreasing inflammation, glucose intolerance, insulin resistance, obesity and type 2 diabetes in both rodents and humans [7-10].

Numerous phytochemical compounds have been studied as potential tools to regulate glucose homeostasis, adipose tissue development and inflammatory tone. Among these, curcumin is widely studied. The dried ground rhizome of the perennial herb *Curcuma longa* is a popular dietary spice in Asia, as used in curry [11]. It is also an integral part of the Indian traditional medicine called *Ayurveda*. The polyphenol curcumin comprises 2-8% of most curcuma preparations and is generally regarded as its most active component, having potent antioxidant, anti-inflammatory, and anticarcinogenic properties [12,13]. Commercial curcumin is readily available as standardized curcuminoid preparations containing 80% curcumin, 15% demethoxycurcumin and 5% bisdemethoxycurcumin [14,15]. Several in vitro studies have demonstrated that curcumin can improve the pattern of markers of inflammation and metabolic disorders related to obesity [12,13,16]. Indeed, studies revealed that curcumin inhibits the activation of the TLR-4 and NF-κB pro-inflammatory signaling pathways in diverse cell types including human adipocytes and macrophages [13,17,18]. Weisberg et al. have demonstrated that oral administration of curcumin ameliorated diabetic status and reduced markers of inflammation in the liver and in the perigonadal adipose tissue of the *ob/ob* mice and mice fed a HF diet. However, the high dose used in this study (3% in the diet) was inconceivable in humans [14]. Other studies have shown that dietary treatment with curcumin improved insulin sensitivity, inflammatory disorders or prevented liver fat accumulation in rodents fed with a HF diet. It is worth noting that the beneficial effects observed in those studies were always demonstrated after a long period of administration (up to 8 weeks) [19-22]. Finally, curcumin treatment for 12 weeks could diminish expansion of adipose tissue and body weight gain probably through inhibition of angiogenesis and adipogenesis in adipose tissue [23,24]. 

The concentration of a plant extract compound that has been used in the study previously mentioned (either in vitro or in vivo in animal models) is a matter of debate. The authors often test doses that are not compatible with the expected reasonable human consumption. Curcumin undergoes an extensive metabolism by the liver and the gut. These phenomena restrain its bioavailability [15,25]. Moreover, due to the long duration of the studies usually found in the literature, it remains difficult to dissociate the direct beneficial effects of the curcumin supplementation from those associated to body weight and fat mass losses. Indeed, a long-term supplementation induces a drastic decrease in body weight and fat mass, that could explain by itself the improvement of metabolism and inflammation. 

While most studies indicate that curcumin glucuronides and tetrahydrocurcumin are less active than curcumin itself, other studies bring controversies concerning the activity of the native curcumin versus its metabolites [15,26]. Therefore, despite curcumin’s multiple medicinal benefits, low oral bioavailability of curcumin continues to be highlighted as a major challenge in developing formulations for clinical efficacy. Piperine, a constituent of pepper, is an inhibitor of hepatic and intestinal glucuronidation. Thus the ingestion of piperine contributes to increase the serum concentration of curcumin and thereby its bioavailability [25]. Moreover, in addition to its bioenhancer properties, piperine would have positive effects such as anti-oxydant or anti-inflammatory and could be considered as a treatment to decrease the cancer cells progression especially via its inhibitory impact on angiogenesis process. 

The purpose of the present study was to determine whether the oral administration of a nutritionally relevant dose of an extract rich in *Curcuma longa* associated with white pepper, to mice could modulate gut microbiota composition and help to offset the HF-induced metabolic disorders and inflammation after only 4 weeks of HF diet. 

## Materials and Methods

### Ethics Statement

The agreement of the animal experiments performed in this study was given by the ethical committee for animal care of the Health Sector of the Université catholique de Louvain, under the supervision of prof. F. Lemaigre et JP Dehoux under the specific number 2010/UCL/MD022. Housing conditions were as specified by the Belgian Law of of 6 April 2010, on the protection of laboratory animals (agreement n° LA 1230314).

### Animals and diet intervention

Twenty four male C57BL/6J mice (9 weeks old at the beginning of the experiment, Charles River Laboratories, France) were housed in groups of 4 per cage in a controlled environment (12-hour daylight cycle) with free access to food and water. After one week of acclimatization, the mice were divided into 3 groups (n=8/group): a control group (CT), fed with a standard diet (CT group), a group fed a HF diet (HF group) and a group fed the same HF diet, supplemented with Curcuma-P®(1.368 g/kg), which contained a mix of *Curcuma longa* extract in order to obtain 0.1 % of curcumin and 0.01% of white pepper in the HF diet (HF-CC group). The composition of the standard diet (AIN93M, Bioserv, Frenchtown, NJ, USA) was the following: 14 g/100 g protein (obtained from casein); 77 g/100 g carbohydrates (obtained from corn starch, maltodextrin 10, sucrose and cellulose BW200) and 4.0 g/100g lipids (obtained from soybean oil). The composition of the HF diet was the following: 26 g/100 g protein (obtained from casein); 20 g/100 g carbohydrates (obtained from maltodextrin 10 and cellulose BW200) and 49 g/100g lipids (obtained from sunflower oil and lard). The caloric value of the diets was 3.85 Kcal/g, 5.88 Kcal/g for CT and HF diets, respectively. Longévie (Froyenne, Belgium) supplied Curcuma-P® ; the composition was 86 % extract of *Curcuma longa*, 9.2% white pepper, 3.4% cellulose, 0.9% silicium dioxide, 0.5% magnesium stearate. Food intake was recorded, taking into account spillage, twice a week during 4 weeks. Body composition was assessed by using a 7.5-MHz time-domain nuclear magnetic resonance (LF50 minispec; Bruker, Rheinstetten, Germany). After 4 weeks of treatment and a 6-hour period of fasting, mice were anesthetised (ketamine/xylazine i.p., 100 and 10 mg/kg, respectively). Blood from cava vein was collected in tubes containing EDTA, then centrifuged (3 min, 13000g, 4°C), and plasma obtained were immediately frozen and stored at -80°C for further analysis. Liver, subcutaneous adipose tissue, ileon, colon and caecum were carefully dissected and weighted before immersion in liquid nitrogen and storage at –80 °C. 

### Microbial analysis of the caecal contents

At the end of the experiment, the total caecum content was collected and weighed before storage at -80°C. Quantitative PCR (Q-PCR) for total bacteria, *Bifidobacterium* spp.*, Lactobacillus* spp. and *Bacteroides-Prevotella* spp. were performed by using Mesa Fast qPCR™ (Eurogentec, Seraing, Belgium). Real-time PCRs were performed with the StepOnePlus™ real time PCR system and software (Applied Biosystems, Den Ijssel, The Netherlands). The primers are detailed in Table S1. Cycle threshold of each sample was then compared with a standard curve (performed in triplicate) made by diluting genomic DNA obtained from BCCM/LMG (Ghent, Belgium) or DSMZ (Braunshweig, Germany) (prior to isolating the DNA, the cell counts were determined by BCCM/LMG, or DSMZ, respectively); five-fold serial dilution of *Bifidobacterium animalis* BCCM/LMG 18900 for *Bifidobacterium* spp., *Bacteroides fragilis* BCCM/LMG 10263 for *Bacteroides-Prevotella* spp. and *Lactobacillus acidophilus* DSM 20079 for *Lactobacillus* spp. and total bacteria 

### Blood parameters

Blood glucose concentration was determined on animals before anesthesia, with a glucose meter (Roche Diagnostic, Meylan, France) on 3.5 µL of blood collected from the tip of the tail vein. Plasma insulin concentrations were determined using an ELISA kit (Mercodia, Uppsala, Sweden). Plasma triglycerides, cholesterol and non-esterified fatty acid concentrations were measured using kits coupling enzymatic reaction and spectrophotometric detection of reaction end-products (Diasys Diagnostic and Systems, Holzheim, Germany). High density lipoprotein cholesterol (HDL-cholesterol) concentration was measured enzymatically after very low density lipoprotein (VLDL), chylomicrons and low density lipoprotein cholesterol (LDL-cholesterol) antibodies precipitation (Diasys Diagnostic and Systems, Holzheim, Germany). The determination of lipid metabolites was based on oxidation, absorbance measurement of quinone imine, which is generated from 4-aminoantypirine and phenol by hydrogen peroxide under the catalytic action of peroxidase (Trinder’s reaction). Finally, LDL-cholesterol was estimated by the Friedewald formula [27].

### Lipid analysis in the liver

Triglycerides and cholesterol were measured in the liver tissue after extraction with chloroform–methanol as described by Neyrinck et al.[28].

### Adipose tissue morphometry

The number of adipocytes per microscopic field (density) was determined on paraffin-embedded hematoxylin-stained eosin- counterstained sections of subcutaneous adipose tissue using the image analyzer software (Motic Image Plus 2.0 ML), as previously described [29].

### Expression of selected genes in tissues

Total RNA was isolated using the TriPure isolation reagent kit (Roche Diagnostics Belgium, Vilvoorde). cDNA was prepared by reverse transcription of 1 µg total RNA using the Kit Reverse transcription System (Promega, Leiden, The Netherlands). Real-time PCRs were performed with the StepOnePlus™ real time PCR system and software (Applied Biosystems, Den Ijssel, The Netherlands) using SYBR Green for detection according to the manufacturer’s instructions. RPL19 RNA was chosen as housekeeping gene. Primer sequences for the targeted mouse genes are summarized in Table S1. All samples were run in duplicate in a single 96-well reaction plate and data were analysed according to the 2-ΔCT method [30]. The identity and purity of the amplified product was checked through analysis of the melting curve carried out at the end of amplification.

### Curcumin and tetrahydrocurcumin analysis

Frozen aliquot of subcutaneous adipose tissues (100-200 mg) were ground and extracted with 1.5 ml of ethyl acetate under stirring for 30 min at room temperature. The solvent extraction step was repeated three times. The pooled extract was evaporated to dryness using a SpeedVac and reconstituted in a methanol-3.6% glacial acetic acid mixture (60:40, v/v). Samples were kept at -20°C for 30 min and centrifuged (5000 g, 30 min, 4°C) [31]. The supernatants were analysed by HPLC using an Agilent 1100 Series HPLC system with a diode array and multiple wavelength detector. Chromatographic separation was achieved on a Nucleodur C18 ec (250 mm x 2 mm, 5 µm) column (Macherey Nagel, Düren, Germany). The mobile phase consisted of acetonitrile-methanol-3.6 % glacial acetic acid (41:23:36, v/v/v) [32]. The system was isocratically run at a flow rate of 0.3 ml/min. The sample detection was achieved at 428 nm for curcumin compounds (curcumin, demethoxycurcumin, bisdemethoxycurcumin) and at 280 nm for tetrahydrocurcumin. The injection volume was 50 µl. Curcumin and tetrahydrocurcumin (Sabinsa, Langen, Germany) in methanol-3.6% glacial acetic acid (60:40, v/v) were used as calibration standards samples. The limits of detection and quantification were evaluated at 20 ng/ml and 60 ng/ml for curcumin (CV=3 %) and at 60 ng/ml and 200 ng/ml for tetrahydrocurcumin (CV=2.5 %).

### Statistical analysis

Results are presented as mean ± SEM and Whiskers plot with minimum and maximum for bacterium. Statistical significance of difference between groups was assessed by one-way analysis of variance (ANOVA) followed by post hoc Tukey’s multiple comparison tests using Graph-Pad Prism (version 5.00 for Windows, GraphPad Software, San Diego, California, USA). Data with different superscript letters are significantly different (p < 0.05).

## Results

### Curcuma-P® supplementation does not affect body weight gain and fat mass development after 4 weeks

**Figure 1:**
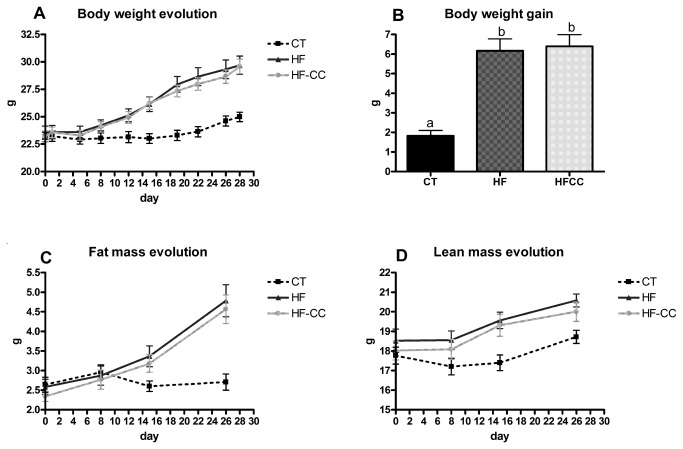
Body weight and body composition. Body weight evolution (A), body weight gain (B), fat mass evolution (C) and lean mass evolution (D) of mice fed a control diet (CT), a high fat diet (HF) or a high fat diet supplemented with Curcuma-P® (HF-CC) for 4 weeks. Data with different superscript letters are significantly different at p<0.05, according to the post hoc ANOVA statistical analysis.

The HF diet induced a huge weight gain (Figure 1 A and B) linked to a significant increase of both fat mass and lean mass as measured by RMN (Figure 1 C and D). This effect was accompanied by fat accumulation in the subcutaneous adipose tissue (table 1). Those parameters were not modified by Curcuma-P® administration. Histological analysis revealed that the adipocyte size in subcutaneous adipose tissue was increased in the HF-fed mice versus controls but was not significantly modified by Curcuma-P® treatment (adipocyte number per field: 343 ± 67, 154 ± 19 and 168 ± 21 for CT, HF and HF-CC groups, respectively, p>0.05 HF-CC versus HF). These results suggest that oral administration of curcumin associated with pepper did not affect adiposity within 4 weeks of treatment. Of note, the weight of the liver, the muscles (gastrocnemius), the spleen or the caecal tissue were not affected by the dietary treatment (table 1). 

**Table 1:** Weight of tissue.

*% body weight*	**CT**	**HF**	**HF-CC**
Liver	3.59 ± 0.07	3.15 ± 0.08	3.51 ± 0.07
Subcutaneous adipose tissue	1.99. ± 0.16**^*a*^**	3.66 ± 0.26**^*b*^**	3.52 ± 0.31**^*b*^**
Spleen	0.27 ± 0.01**^*a*^**	0.24 ± 0.01^ab^	0.23 ± 0.01**^*b*^**
Gastrocnemius	1.14 ± 0.02**^*a*^**	1.03 ± 0.02**^*b*^**	1.01 ± 0.02**^*b*^**
Caecal tissue	0.19 ± 0.01	0.17 ± 0.01	0.18 ± 0.01

Mice were fed a high fat diet (HF) or a high fat diet supplemented with Curcuma-P® (HF-CC) for 4 weeks. Data with different superscript letters are significantly different at p<0.05, according to the post hoc ANOVA statistical analysis.

### Curcuma-P® supplementation does not change the metabolic status of mice after 4 weeks

Supplementation with Curcuma-P® did not improve glucose or lipid homeostasis after 4 weeks of HF diet (table 2). Indeed, there was no difference in fasting hyperglycemia, fasting insulinemia or insulino-resistance index (HOMA). In the same way, the serum lipids (triglycerides, non esterified fatty acids, total cholesterol, LDL and HDL-cholesterol) were not modulated under HF-CC diet. Interestingly, Curcuma-P® addition in the HF diet tended to decreased the total cholesterol content in the liver (p=0.1). However, mRNA levels of markers controlling the hepatic cholesterol trafficking (ABCA1, ABCG5 and ABCG8) were not significantly modified by the dietary treatment (data not shown).

**Table 2:** Blood and hepatic parameters.

	**CT**	**HF**	**HF-CC**
*Serum (mM)*			
fasting glycemia	6.75 ± 0.18**^*a*^**	8.97 ± 0.28**^*b*^**	8.91 ± 0.19**^*b*^**
fasting insulinemia	82 ± 20	94 ± 28	103 ± 25
insulin resistance index	24 ± 5	38 ± 11	40 ± 9
triglycerides	0.53 ± 0.05	0.65 ± 0.08	0.67 ± 0.07
non esterified fatty acids	0.49 ± 0.04	0.49 ± 0.03	0.51 ± 0.03
total cholesterol	1.89 ± 0.09**^*a*^**	2.42 ± 0.07**^*b*^**	2.58 ± 0.05**^*b*^**
LDL-cholesterol	0.46 ± 0.05**^*a*^**	0.81 ± 0.03**^*b*^**	0.86 ± 0.06**^*b*^**
HDL-cholesterol	1.19 ± 0.05**^*a*^**	1.32 ± 0.03^ab^	1.41 ± 0.04**^*b*^**
*Liver lipid content (nmol/mg protein)*			
triglycerides	80 ± 7	76 ± 7	72 ± 6
total cholesterol	78 ± 10	79 ± 7	54 ± 9

Mice were fed a high fat diet (HF) or a high fat diet supplemented with Curcuma-P® (HF-CC) for 4 weeks. LDL, low density liprotein; HDL, high density lipoprotein. Data with different superscript letters are significantly different at p<0.05, according to the post hoc ANOVA statistical analysis.

### Curcuma-P® supplementation decreases HF-induced expression of inflammatory markers in the subcutaneous adipose tissue without affecting inflammation in the gastrointestinal tract

**Figure 2:**
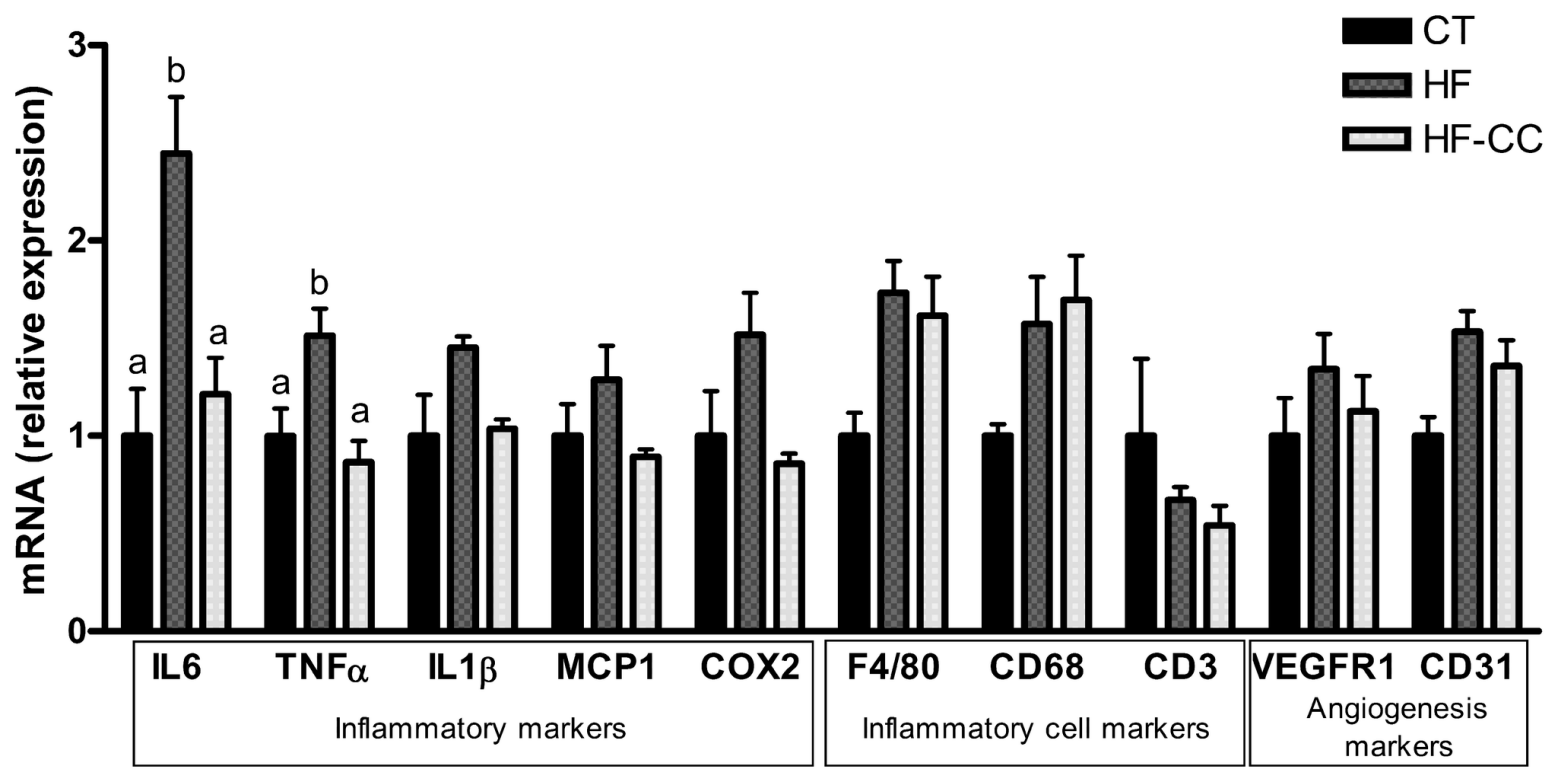
mRNA levels of key markers in subcutaneous adipose tissue. Mice were fed a high fat diet (HF) or a high fat diet supplemented with Curcuma-P® (HF-CC) for 4 weeks. Values are expressed relative to controls (set at 1). Data with different superscript letters are significantly different at p<0.05, according to the post hoc ANOVA statistical analysis.

The co-supplementation with curcuma extract and white pepper significantly down-regulated the main HF-induced pro-inflammatory cytokines (IL6 and TNFα) in the subcutaneous adipose tissue (Figure 2). The expression of chemo-attractant protein-1 (MCP1), the inducible cyclooxygenase 2 (COX-2) producing prostaglandins (PGE_2_) and IL1β were non significantly decreased after Curcuma-P® supplementation. The higher expression of CD68 and F4/80, following HF treatment, which are markers of inflammatory cell infiltration, was not affected by the addition of Curcuma-P®. It is interesting to note that Curcuma-P® supplementation did not modify the expression of main angiogenesis markers (VEGFR1 and CD31) in the subcutaneous adipose tissue (Figure 2). Curcuma-P® supplementation did not affect pro-inflammatory markers in other tissues, such as the ileon, the colon and the liver (table 3).

**Table 3:** Gene expression levels of key pro inflammatory markers in different parts of gut and in the liver.

	**CT**	**HF**	**HF-CC**
*Ileon*			
IL1β	1.000 ± 0.072	1.010 ± 0.120	1.238 ± 0.174
IL6	1.000 ± 0.136	1.466 ± 0.203	1.443 ± 0.256
TNFα	1.000 ± 0.116	1.263 ± 0.064	1.405 ± 0.159
MCP-1	1.000 ± 0.175	0.946 ± 0.146	1.161 ± 0.211
COX-2	1.000 ± 0.101	0.705 ± 0.086	0.748 ± 0.055
*Colon*			
IL1β	1.000 ± 0.067	1.138 ± 0.338	1.078 ± 0.097
IL6	1.000 ± 0.163	1.293 ± 0.326	1.628 ± 0.450
TNFα	1.000 ± 0.127	0.952 ± 0.116	1.200 ± 0.172
MCP1	1.000 ± 0.100	1.004 ± 0.318	1.135 ± 0.141
COX2	1.000 ± 0.061	1.099 ± 0.179	1.095 ± 0.073
*Liver*			
IL1β	1.000 ± 0.114	1.303 ± 0.363	1.338 ± 0.154
IL6	1.000 ± 0.231	0.898 ± 0.211	1.124 ± 0.185
TNFα	1.000 ± 0.067	1.109 ± 0.114	1.412 ± 0.212
MCP-1	1.000 ± 0.113	1.128 ± 0.194	1.417 ± 0.259

Mice were fed a high fat diet (HF) or a high fat diet supplemented with Curcuma-P® (HF-CC) for 4 weeks. Values are expressed relative to controls (set at 1). ANOVA test, p>0.05

### Curcuma-P® supplementation does not modify the gut bacteria known to impact inflammation

**Figure 3:**
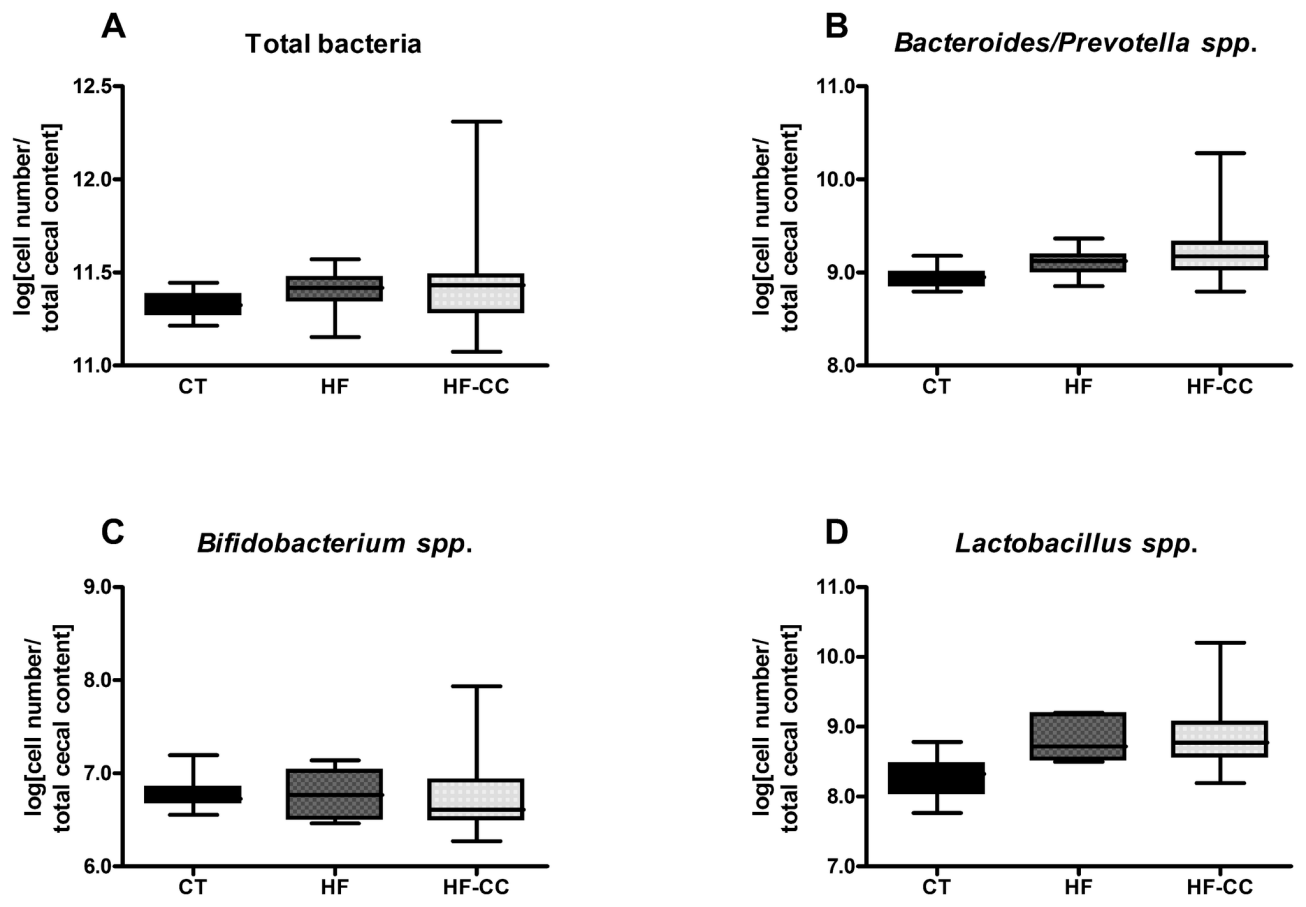
Bacterial quantification per total caecal content. Caecal bacterial content of total bacteria (A), *Bacteroides*-*Prevotella* spp. (B), *Bifidobacterium* spp. (C), *Lactobacillus* spp. (D). Bacterial quantities are expressed as Log_10_ (bacterial cells/caecal content wet weight). Mice were fed a high fat diet (HF) or a high fat diet supplemented with Curcuma-P® (HF-CC) for 4 weeks. ANOVA test, p>0.05.

The Curcuma-P® supplementation did not change the content in total bacteria in the caecal content, measured by PCR after 4 weeks of HF diet (Figure 3). Moreover, this treatment did affect neither the main Gram positive species able to control the systemic inflammation (*Bifidobacterium* spp. and *Lactobacillus* spp.) nor the main Gram negative bacteria (*Bacteroides/prevotella* spp.) that may release pro-inflammatory lipopolysaccharides (LPS).

### Tetrahydrocurcumin, one of the main metabolites of curcumin, accumulates in the adipose tissue after Curcuma P® supplementation

HPLC analysis revealed the presence of tetrahydrocurcumin (235 ± 78 ng/ 100 mg tissue) and the absence (<LOQ) of curcumin inside the subcutaneous adipose tissue after 4 week of Curcuma-P® administration. Curcumin and its metabolite were undetectable (<LOD) in the adipose tissue of the CT mice or the HF mice. Furthermore, neither tetrahydrocurcumin nor curcumin were detected in the plasma of HF-CC mice. 

## Discussion

The health promoting effect of plant constituents and extracts is increasingly gaining interest, a phenomenon explaining that their consumption is on the rise in the western world [33-35]. The health benefits of curcumin consumption have received considerable scientific focus. Evidence from in vivo studies in HF-fed mice demonstrated that curcumin supplementation (0.05%) for 12 weeks can reduce body weight gain, adiposity without affecting food intake [23]. In another study, it was shown that oral administration of curcumin at high dose (3% in the diet) ameliorated HF-induced diabetes as determined by glucose and insulin tolerance testing [14]. In our study, Curcuma-P® did not significantly modify neither body weight, nor HF-induced fat mass accumulation nor adipocyte size, after 4 weeks of dietary treatment. Moreover, glycemia, insulinemia and the insulin resistance index (HOMA) values were not affected by the co-supplementation suggesting that the improvement in glucose homeostasis found in longer study could be mainly attributed to fat mass and body weight losses. In long term studies (up to 8 weeks), authors have demonstrated that curcumin lowers triglycerides, cholesterol and free fatty acids in the plasma or the liver of HF-fed hamsters or rats [16,19,22]. A lower activity of hepatic enzymes, such as HMG-CoA reductase and Acyl CoA cholesteryl acyl transferase (ACAT), was demonstrated by Jang et al. in HF-fed hamsters or a higher hepatic cholesterol catabolism, and by Babu & Srinivasan in streptozotocin-induced diabetic rats [22,36]. In our study, we only observed a downward trend in the cholesterol content in the liver, but we were unable to demonstrate serum lipid lowering properties (triglycerides, free fatty acids, cholesterol).

Despite the minor impact on lipid homeostasis and the lack of effect on adiposity, we have shown that administration of *Curcuma longa* extract associated with pepper decreased the expression of two important genes (IL6 and TNFα) involved in the induced-HF proinflammatory tone in the subcutaneous adipose tissue, leading back to values similar to the ones observed in animals receiving a standard diet. We also observed that the expression of others proinflammatory markers such as IL1β and MCP1, and of COX2, has a similar trend to decrease upon Curcuma-P® supplementation. This effect was relatively tissue-specific since the expression of the same markers was not affected by Curcuma-P® in the liver, the ileon and the colon. 

The anti-inflammatory effect of the extract in the adipose tissue is independent of the extent of inflammatory cells infiltration in the adipose tissue, which was promoted by the HF diet. Indeed, the expression of pro-inflammatory cell markers (F4/80 and CD68), signing macrophage infiltration which is promoted by the HF diet, was not modified by the supplementation with Curcuma-P®. In addition, CD3 mRNA expression that relates to lymphocyte infiltration was identical in HF and HF-CC animals. Others studies have already demonstrated the anti-inflammatory properties of both curcumin and piperine, the active compound of white pepper. In 3T3-L1 adipocytes, Shehzad et al (2010) showed that curcumin decrease the expression of various pro-inflammatory cytokines such as IL1, IL6 or TNFα and COX2 gene expression [13,18]. These anti-inflammatory effects were linked to the inhibition of the NF-κB signalling pathway. In fact, the authors highlighted that curcumin specifically inhibit the IKK signalling complex involved in the phosphorylation of IκB leading to the inhibition of the NF-κB signalling pathway. In view of these results, we postulate that the anti-inflammatory effect of the curcuma extract used in the present study could be dependent on the same mechanisms. 

Several studies demonstrated anti-angiogenic properties of curcumin associate with piperine which are essential for their action against the cancer development and which could be proposed as an explanation its capability to limit the adipose tissue expansion, at least for curcumin [23,37,38]. However, the main angiogenesis markers (VEGFR1 and CD31) were not modified in the subcutaneous adipose tissue after curcuma supplementation in our conditions.

Curcumin metabolism in the intestine involves sulfation, glucuronidation, and reduction reactions, which result in poor intestinal absorption (for review see 12,15). These metabolites have a very short half-life and do not exhibit sufficient lipophilicity for cellular uptake. Consequently, to improve the systemic absorption of curcumin is becoming a real challenge. When co-supplemented with piperine (an alkaloid present pepper), an important increase was found in the absorption of curcumin [12]. In the present study, although our curcuma extract was associated with white pepper, we did not found measurable levels of curcumin in the serum or in the adipose tissue. Tetrahydrocurcumin was found to show better antidiabetic and antioxidant activity than curcumin in type 2 diabetic rats [39]. Two studies established anti-inflammatory activities of tetrahydrocurcumin [40,41]. In the present study, we observed substantial levels of tetrahydrocurcumin in the subcutaneous adipose tissue suggesting that this metabolite accumulated inside white adipose tissue and might be the metabolite responsible of the anti-inflammatory effect observed after administration of *Curcuma longa* added in the HF diet. This hypothesis merits further investigations.

The intestine harbors a complex microbial ecosystem comprising a considerable metabolic versatility, and is increasingly considered as a symbiotic partner for the maintenance of health [42]. Indeed, several data suggest that the activity of the gut microbiota is a factor to take into account when assessing the risk factors related to obesity and associated inflammatory disorders [3-6]. It is also known that phenolic components of common foods readily contribute to gut bacteria modulation [43-45]. Interestingly, the number of bifidobacteria was inversely correlated with the development of fat mass, glucose intolerance, adipose tissue inflammation and LPS level [7,10,30,46]. This species as well as other species known to impact inflammation (*Lactobacillus spp.* and *Bacteroides* *spp.*) were not affected by the co-supplementation of curcuma extract associated with pepper, thereby suggesting that the anti-inflammatory effect of this co-supplementation on the adipose tissue is not related in our protocol, to changes of bacteria prone to have anti- or pro-inflammatory properties. The capacity of the gut microbiome, encoded by the collective genomes of the gut microbiota or gut metagenome, includes the metabolism of indigestible polyphenols derived from fruit and plant [26,45]. Therefore, we cannot exclude a role of gut microbiota on curcumin metabolism, since curcumin-converting microorganisms can be isolated from human feces. The microbial metabolism is able to convert curcumin into tetrahydrocurcumin and, as shown in the present study, this metabolite may reach the adipose tissue [47]. The relative contribution of host tissue versus microbiota in curcumin metabolism in vivo could be considered as an interesting perspective of this work.

In conclusion, the present study shows that oral administration of *Curcuma longa* associated with white pepper at doses that can be extrapolated to human exposure upon supplementation improves pro-inflammatory disorders in the subcutaneous tissue following HF diet feeding. This effect was the first event, occurring before any change in adiposity and independent of the expression of angiogenic markers. Although further studies are needed in order to determine whether tetrahydrocurcumin is the main metabolite responsible for these effects, and how it is produced in the body, our results suggest that *Curcuma longa* extract associated to white pepper can confer positive health impacts and might be a natural alternative in the prevention of inflammatory disorders occurring early in the development of obesity upon HF feeding

## Supporting Information

Table S1 **Sequence of primers used for real-time PCR.**
(DOCX)
