# Supplementary material for: Curcuma longa Extract Associated with White Pepper Lessens High Fat Diet-Induced Inflammation in Subcutaneous Adipose Tissue
Source: PLoS One. 2013 Nov 19;8(11):e81252. doi: 10.1371/journal.pone.0081252 (PMC3834320; doi:10.1371/journal.pone.0081252)
Supplement: Table S1 — Sequence of primers used for real-time PCR. (DOCX) [file pone.0081252.s001.docx]

Supplemental Table S1: Sequence of primers used for real-time PCR

| *Gene* | *Primer Forward* | *Primer Reverse* |
| --- | --- | --- |
|  |  |  |
| **Inflammation** |  |  |
| IL1β | TCGCTCAGGGTCACAAGAAA | CATCAGAGGCAAGGAGGAAAAC |
| IL6 | ACAAGTCGGAGGCTTAATTACACAT | TTGCCATTGCACAACTCTTTC |
| TNFα | AGCCCCCAGTCTGTATCCTT | GGTCACTGTCCCAGCATCTT |
| MCP1 | GCAGTTAACGCCCCACTCA | CCCAGCCTACTCATTGGGATCA |
| F4/80 | TGACAACCAGACGGCTTGTG | GCAGGCGAGGAAAAGATAGTGT |
| CD68 | CTTCCCACAGGCAGCACAG | AATGATGAGAGGCAGCAAGAGG |
| CD3 | GCAAGAATAGGAAGGCCAAG | GGTCCACAGAAGGCGATGT |
| COX2 | TGACCCCCAAGGCTCAAATAT | TGAACCCAGGTCCTCGCTTA |
|  |  |  |
| **Angiogenesis process** |  |  |
| VEGFR | TGGCCAGAGGCATGGAGT | TCGCAAATCTTCACCACATTG |
| CD31 | GGAACGAGAGCCACAGAGAC | TGCACTGCCTTGACTGTCTT |
|  |  |  |
| **Microbiota** |  |  |
| Total bacteria | ACTCCTACGGGAGGCAGCAG | ATTACCGCGGCTGCTGG |
| *Bifidobacterium spp* | TCGCGTCYGGTGTGAAAG | CCACATCCAGCRTCCAC |
| *Bacteroides-Prevotella spp* | TCGCGTCYGGTGTGAAAG | CGGAYGTAAGGGCCGTGC |
| *Lactobacillus spp* | CCTTTCTAAGGAGCGAAGGAT | AATTCTCTTCTCGGTCGCTCTA |
